# APC/C – the master controller of origin licensing?

**DOI:** 10.1186/1747-1028-2-8

**Published:** 2007-02-23

**Authors:** Umasundari Sivaprasad, Yuichi J Machida, Anindya Dutta

**Affiliations:** 1 Department of Biochemistry and Molecular Genetics, University of Virginia School of Medicine, Charlottesville, VA 22908, USA

## Abstract

DNA replication must be tightly controlled to prevent initiation of a second round of replication until mitosis is complete. So far, components of the pre-replicative complex (Cdt1, Cdc6 and geminin) were considered key players in this regulation. In a new study, Machida and Dutta have shown that depletion of Emi1 caused cells to replicate their DNA more than once per cell cycle [1]. This effect was dependent on the ability of Emi1 to inhibit the APC/C. In addition to its role in regulating entry into mitosis, oscillation of APC/C activity regulates pre-RC formation: high APC/C activity in late M/G1 allows pre-RC formation and low APC/C activity in S/G2 prevents pre-RC formation for a second time thereby preventing rereplication. Each redundant pathway to prevent rereplication is dependent on regulating one of the pre-RC components, and all of the pathways are co-regulated by Emi1 through the APC/C. In this commentary we discuss how this new role of Emi1 adds to our understanding of the regulation of replication initiation. We also review the literature to analyze whether APC/C has a role in regulating endoreduplication (a normal state of polyploidy in some differentiated cells). Similarly a role of premature APC/C activation in genomic instability of tumors is discussed.

## Background

DNA replication must be restricted to once per cell cycle and must be followed by mitosis to yield two cells each with the same amount of DNA. Hence replication is a tightly regulated process with many redundant pathways in place to prevent uncontrolled DNA synthesis (rereplication) and to ensure that each daughter cell receives a complete complement of chromosomes. To understand how replication is regulated we must first understand the factors that are involved in the process and how replication is controlled during the normal cell cycle.

The cell cycle describes the four stages of cell division. Specific enzymes called cyclin dependent kinases (cdks) along with their associated proteins (cyclins) regulate progression through the cell cycle. Cdk activity is tightly controlled by rapid synthesis and degradation of the associated cyclin. The first phase is G1 where the cells prepare to synthesize new DNA. Cyclin D/cdk4 activity increases early in G1 and this results in increased cyclin E/cdk2 activity late in G1 that promotes entry into the S phase. DNA synthesis is completed in the S phase and proper completion of this step is dependent on cyclin A/cdk2. In G2, cells prepare for division followed by M phase where the cells undergo mitosis. Cyclin A/cdk1 and cyclin B/cdk1 activity is required for G2 and M phase progression. Mitosis results in two daughter cells with a complete copy of the genetic material from the original cell.

The initiation of replication and its regulation is an area of active study and there are numerous excellent reviews on this subject [2-4]. Our current understanding of pre-RC formation is briefly summarized below. A six-subunit complex called ORC (origin recognition complex) binds to discrete regions of the chromosome called origins of replication. The binding of two proteins Cdt1 and Cdc6 follows, and they in turn recruit the MCM2-7 complex to the origin. The MCM2-7 complex is the putative replicative helicase that unwinds the DNA and allows downstream factors access to the DNA to initiate replication. The ORC, Cdt1, Cdc6, MCM2-7 complex is called the pre-RC (pre-replicative complex). Formation of the pre-RC occurs in the late M and G1 phases of the cell cycle. In late G1 and S phase, as cyclin E and cyclin A levels increase, the associated cdks phosphorylate and recruit downstream factors to the origins followed by association of DNA polymerases to initiate new DNA synthesis.

Once the MCM complex is recruited to the origins, a second round of replication initiation is prevented by multiple mechanisms. Levels of geminin, a Cdt1 inhibitor, increase as cells enter S phase. Geminin associates with Cdt1 and prevents MCM loading on the chromatin. Cdc6 is phosphorylated by cdks and is translocated out of the nucleus. A portion of Cdt1 is also phosphorylated by cdk2 and targeted for degradation. The end result is prevention of MCM2-7 complex loading on the chromatin until the cells have completed mitosis. At the end of mitosis geminin levels decrease, cdk activity decreases and, as the cells enter G1, pre-RC formation begins again.

Degradation of Cdt1, Cdc6, geminin and the cyclins is mediated by two well-coordinated ubiquitin ligases. They are the SCF (Skp1/Cul1/F-box) and the APC/C (anaphase promoting complex/cyclosome) complexes (reviewed in [5]). Among other proteins, the SCF complex regulates the level of the G1 cyclins, targeting them for degradation once cells enter S phase. One of several F-box proteins (the substrate recognition component of the SCF), Fbw7, binds to cyclin E and catalyzes its ubiquitination, targeting the cyclin for proteasomal degradation. The APC/C complex has two substrate recognition components – Cdc20 and Cdh1. Each APC/C complex contains either Cdc20 or Cdh1. This complex is active in the M (APC/C^Cdc20^) and late M/G1 (APC/C^Cdh1^) phases of the cell cycle. The repertoire of proteins that the APC/C complex targets for degradation includes the S and M phase cyclins (A and B), Cdc6, and geminin.

Emi1 is a negative regulator of APC/C activity (early mitotic inhibitor 1). Normally Emi1 levels are controlled by two different mechanisms. Polo-like kinase 1 (Plk1) phosphorylates Emi1 and targets it for degradation by the SCF^βTRCP ^complex early in mitosis [6-9]. Emi1 also associates with a protein called Evi5 [10]. Evi5 binds to Emi1 close to the Plk1 phosphorylation site and protects Emi1 from being phosphorylated and subsequently degraded. In S phase, as Plk1 levels increase, it targets Evi5 for degradation. This exposes the Plk1 phosphorylation site on Emi1. Phosphorylation of Emi1 triggers its degradation early in mitosis, finally activating the APC/C.

Rereplication occurs when pathways controlling pre-RC formation are disrupted. In mammalian cells, depletion of geminin or overexpression of Cdt1 is sufficient to cause rereplication [11-14]. In yeast, premature or constitutive cdk inactivation is sufficient to cause rereplication (reviewed in [2]). Whether cdk inactivation (in addition to disruption of the geminin-Cdt1 balance) is required for rereplication in mammalian cells has never been clearly established. However inhibition of cdk activity alone did cause rereplication in some cell lines [15,16]. A recent study by Machida and Dutta [1] has identified Emi1 as an upstream regulator of both the geminin-Cdt1 and cyclin A-cdk pathways. Emi1 depletion triggers rereplication in mammalian cells and this is mediated by degradation of geminin and inhibition of cyclin A associated kinase activity. Depletion of geminin releases its inhibitory effects on pre-RC formation allowing Cdt1 to remain active on the chromatin. Depletion of cyclin A prevents the cyclin A/cdk complex from phosphorylating Cdt1, Cdc6 and MCMs, leaving them all active and capable of reforming the pre-RC even after the first round of replication has begun. This then allows the origins to fire for a second and possibly additional rounds of replication. In HCT116 cells, maintaining the geminin-Cdt1 balance alone is sufficient to prevent rereplication. This is consistent with studies in Drosophila where depletion of either geminin or cyclin A can induce rereplication [17,18]. In HeLa cells, however, cdk activity must additionally be inhibited to induce rereplication [1]. Overexpressing a non-degradable form of cyclin A was sufficient to prevent rereplication after Emi1 depletion. This suggests that either of the two pathways is sufficient for preventing rereplication. This study also highlights the importance of high cdk activity. Like the yeast, high cdk activity in HeLa cells is sufficient to prevent rereplication. However, higher eukaryotes have evolved additional levels of control (like geminin) to prevent rereplication. Ultimately, both pathways are regulated by the APC/C and both pathways prevent rereplication in S and G2 phases of the cell cycle by preventing pre-RC formation. While the role of Emi1 in regulating entry into S and M phases of the cell cycle is well established, this study reveals its new role as a key regulator of pre-RC formation.

In HCT116 cells, geminin depletion is sufficient to cause rereplication [1,11,12]. However in HeLa cells, cyclin A levels must also decrease [1]. It is important to investigate why the cdk-dependent pathway is ineffective in preventing rereplication in some cancer cells like HCT116. One possibility is that the cyclin A-cdk pathway may already be disrupted in HCT116 cells. For example, the levels of the proteins targeted by the cyclin A/cdk pathway (either Cdt1 or Cdc6) may be elevated in these cells, rendering them less sensitive to control by cyclin A/cdk. This possibility is consistent with the observation that overexpression of Cdt1 and Cdc6, or a non-degradable form of Cdt1 alone can trigger rereplication in cancer cells [13,14,19] though additional work is necessary to confirm this model.

### APC/C oscillation regulates pre-RC formation

**Figure 1:**
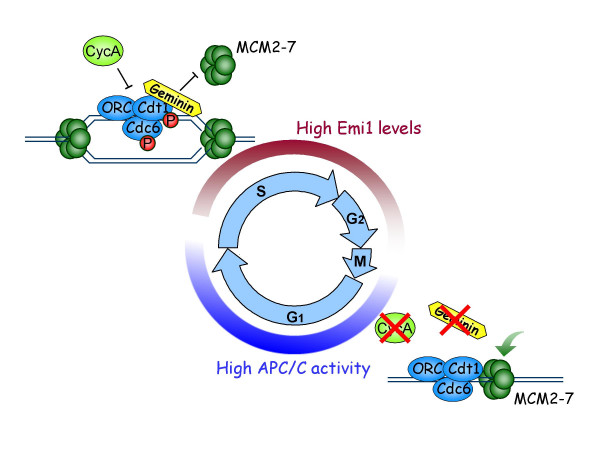
**APC/C oscillation regulates pre-RC formation**. In G1, APC/C activity is high and prevents accumulation of cyclin A and geminin, thereby allowing pre-RC formation. At the G1/S transition, Emi1 levels increase, inhibiting the APC/C. This allows the accumulation of cyclin A and geminin. Geminin associates with Cdt1 and prevents recruitment of MCM to the origins. Cyclin A/cdk phosphorylates Cdc6 (which translocates it out of the nucleus) and Cdt1 (which targets it for degradation by the proteasome). As cells enter G2, Emi1 is degraded, activating the APC/C. The APC/C then degrades cyclin A and geminin as cells enter the next cell cycle.

Tight control of APC/C activity is important for proper pre-RC formation (Figure 1). Emi1 levels increase at the G1/S transition. This inactivates the APC/C allowing the accumulation of cyclin A and geminin [20,21]. Cyclin A associates with the appropriate cdk and phosphorylates Cdc6, Cdt1 and other proteins. This degrades Cdt1 and translocates Cdc6 out of the nucleus preventing pre-RC assembly and thereby a second round or replication. Geminin associates with the remaining Cdt1 preventing it from recruiting additional MCM complexes to the origins. Therefore low APC/C activity in S and G2 phases of the cell cycle is important to prevent rereplication.

In contrast high APC/C activity is important in G1 to promote pre-RC formation. The APC/C degrades the mitotic cyclins and geminin. Degrading the cyclins keeps cdk activity low in G1. This in turn releases the inhibition of Cdc6 and allows Cdt1 accumulation, promoting pre-RC formation. Indeed, Mcm4 loading on the chromatin was inhibited in cells overexpressing cyclin E, and could be restored by inhibiting cdk activity [22]. Geminin degradation also allows Cdt1 to associate with the MCM complex, allowing cells to reestablish the pre-RC for the next round of replication. A previous study has shown that overexpression of a non-degradable form of geminin prevented Mcm2 loading on the chromatin and consequently origin licensing [23]. Therefore APC/C oscillation governs pre-RC formation by regulating Cdt1-geminin balance and cdk activity. Emi1, by virtue of its role as the APC/C inhibitor, plays a crucial role in preventing rereplication by stabilizing geminin and cyclin A.

### APC/C and endoreduplication

While rereplication is strictly prevented in the normal cell cycle, this strict control over DNA replication is selectively inactivated during a normal developmental process called endoreduplication. In humans, this is seen in cells of the liver and pancreas, trophoblast cells of the placenta and megakaryocytes [24,25]. During their development/maturation, these cells undergo repeated rounds of DNA synthesis without an intervening mitosis event. Endoreduplication is also observed in numerous plant cells [26], along with the follicle cells [27] and salivary glands of Drosophila [28]. If the APC/C regulates pre-RC formation, the APC/C may also regulate endoreduplication. Indeed, there are several lines of evidence from plants, Drosophila, and mammalian cells to support this hypothesis.

In plants, cyclin A levels decreased once cells began endoreduplication and limiting cyclin A degradation prevented endoreduplication [29]. Levels of polyploidy 1, a repressor of cyclin A, are increased in some endoreduplicating Arabidopsis cells and overexpressing this protein is sufficient to induce endoreduplication [30]. Finally, several studies have shown that Ccs52A, the ortholog of Cdh1 (the subunit of APC/C) is essential for endoreduplication (reviewed in [26]). In one study, the authors demonstrated that cells containing antisense oligos to Ccs52A did not differentiate into polyploid nitrogen-fixing nodules [31]. While activating the APC/C could be important for endoreduplication, the role of Emi1 in endoreduplication of plant cells cannot be determined because there are no known orthologs of Emi1 in plants.

In Drosophila, fizzy related (fzr), the ortholog of Cdh1 is required for endoreduplication [28]. In this study, fzr deficient embryos show different phenotypes based on the cell type. In epidermal cells, fzr deletion triggers rereplication but in cells of the salivary gland, fzr deletion prevents endoreduplication. This suggests a cell type specific role for fzr. Deletion of Rca1, the homolog of Emi1 in Drosophila also yielded cells with giant nuclei and increased DNA content [32]. While the authors propose that Rca1 may be important for endoreduplication, this has not yet been established experimentally.

In mammalian cells a direct role of the APC/C in mammalian endoreduplication has yet to be confirmed. One clue comes from the study of Emi1 knockout mice [33]. The absence of Emi1 was embryonic lethal. During development of Emi1-/- embryos, the trophoblast cells continued to endoreduplicate like the normal embryo while the inner cell mass could not proliferate and eventually underwent apoptosis. This suggests that Emi1 has a role during the normal cell cycle. However the persistence of the trophoblast cells in the Emi1-/- embryos suggests that Emi1 levels may naturally be low in endoreduplicating cells. More indirect evidence for the role of the APC/C in endoreduplication comes from studies on APC/C substrates. Decrease in cyclin B (instead of cyclin A) is required to initiate endoreduplication in megakaryocytes [34]. Cyclin B is also an APC/C substrate and this is consistent with a role for APC/C activation in endoreduplication. Recently, researchers attempted to generate a geminin knockout mouse [35]. This study showed that homozygous geminin deletions were embryonic lethal. The egg, after reaching the eight-cell stage, underwent premature endoreduplication and developed trophoblast properties without forming any inner cell mass cells required for formation of the embryo. The common theme in these studies is the importance of regulating both the Cdt1-geminin pathway and the cyclin/cdk pathway, both of which are, in turn, regulated by the APC/C.

In normal cells rereplication activates the DNA damage checkpoint preventing further DNA replication [36]. Consistent with this, Emi1 depletion activated the DNA damage checkpoint [1]. It is not known whether such checkpoints are active or inhibited in endoreduplicating cells. The interplay between endoreduplication and checkpoint activation during development remains to be understood. While this research strongly corroborates what is already known about the role of APC/C in endoreduplication, there are more questions than answers at this time regarding this potential role for Emi1 in endoreduplication and more research is needed to find the answers.

### Implications of rereplication in genomic instability

The significance of two redundant pathways to prevent rereplication remains to be better understood. Cells might have developed two overlapping pathways so that even if one fails the other pathway can regulate pre-RC function. For example, even if gemininis depleted and cannot inhibit Cdt1, cyclin A associated cdk2 can phosphorylate Cdt1 and target it for degradation, thereby preventing rereplication. Emi1, however, co-regulates both pathways and loss of Emi1 causes complete loss of replication control. This could result in genomic instability, a hallmark of cancers. Genomic instabilities, such as large-scale insertions, deletions, translocations, polyploidy and aneuploidy, are frequently observed when proteins involved in DNA damage checkpoints, DNA replication, and the cell cycle are affected [37]. Loss of Emi1 could therefore be another trigger for genomic instability.

While loss of both pathways (e.g. by Emi1 depletion) cause large-scale rereplication, loss of either geminin or cyclin A may increase the frequency of micro-rereplication – rereplication of short segments of DNA that cannot be easily detected by most assays currently in use. Such spontaneous re-firing of origins could result in genomic instability and promote tumorigenesis. This is consistent with the observation that Cdt1 and Cdc6 levels are upregulated in small cell lung carcinomas [38] and that Cdt1 overexpression could cause chromosome damage in fibroblasts [39].

Loss of APC/C regulation has been implicated in cancers. The Evi5 chromosomal locus is frequently targeted for recombination in several cancers and a truncated Evi5 is expressed in a patient with neuroblastoma (reviewed in [40]). Loss of Evi5 will destabilize Emi1 by triggering its phosphorylation by Plk1. Numerous studies have also linked Plk1 overexpression to cancers [41]. Plk1 overexpression can trigger Evi5 and Emi1 degradation and activate the APC/C [9,10]. All these pathways, by indirectly decreasing Emi1 levels and activating the APC/C, could ultimately result in genomic instability. Although no studies so far have directly linked decreased levels of Emi1 the genomic instability of cancers, this possibility and should be addressed experimentally.

In summary, the recent study by Machida and Dutta highlights the significance of strict control of APC/C activity throughout the cell cycle. The APC/C controls two redundant pathways to prevent rereplication. Improper activation of the APC/C by depleting the APC/C inhibitor Emi1 disrupts S phase inhibition of pre-RC activity and allows rereplication. While there is evidence to suggest that APC/C activity is critical for normal processes like endoreduplication, and loss of APC/C regulation may be important for tumorigenesis, the direct involvement of the APC/C in both these processes remains to be established.

## Competing interests

The author(s) declare that they have no competing interests.
